# From causal loop diagrams to future scenarios: Using the cross-impact balance method to augment understanding of urban health in Latin America

**DOI:** 10.1016/j.socscimed.2021.114157

**Published:** 2021-06-21

**Authors:** Ivana Stankov, Andres Felipe Useche, Jose D. Meisel, Felipe Montes, Lidia MO. Morais, Amelia AL. Friche, Brent A. Langellier, Peter Hovmand, Olga Lucia Sarmiento, Ross A. Hammond, Ana V. Diez Roux

**Affiliations:** a Urban Health Collaborative, Dornsife School of Public Health, Drexel University, 3600 Market St, Philadelphia, PA, 19104, USA; b South Australian Health and Medical Research Institute, North Terrace, Adelaide, SA, 5000, Australia; c Department of Industrial Engineering, Universidad de Los Andes, Bogotá, Colombia; d Social and Health Complexity Center, Universidad de Los Andes, Bogotá, Colombia; e Facultad de Ingeniería, Universidad de Ibagué, Carrera 22 Calle 67, Ibaguè, 730001, Colombia; f Observatory for Urban Health in Belo Horizonte, Belo Horizonte, Brazil; g School of Medicine, Federal University of Minas Gerais, Belo Horizonte, Brazil; h Department of Health Management and Policy, Dornsife School of Public Health, Drexel University, 3215 Market St, Philadelphia, PA, 19104, USA; i Center for Community Health Integration, Case Western Reserve University, Cleveland, OH, USA; j Department of Public Health, School of Medicine, Universidad de Los Andes, Bogotá, Colombia; k Brown School at Washington University in St. Louis, One Brookings Drive, St Louis, MO, 36130, USA; l Center on Social Dynamics and Policy, The Brookings Institution, 1775 Massachusetts Ave NW, Washington, DC, 20036, USA; m Santa Fe Institute, 1399 Hyde Park Rd, Santa Fe, NM, 87501, USA

**Keywords:** Urban health, Complex system, Cross-impact balance, Chronic disease, Diet, Transportation, Latin America

## Abstract

Urban health is shaped by a system of factors spanning multiple levels and scales, and through a complex set of interactions. Building on causal loop diagrams developed via several group model building workshops, we apply the cross-impact balance (CIB) method to understand the strength and nature of the relationships between factors in the food and transportation system, and to identify possible future urban health scenarios (i.e., permutations of factor states that impact health in cities).

We recruited 16 food and transportation system experts spanning private, academic, non-government, and policy sectors from six Latin American countries to complete an interviewer-assisted questionnaire. The questionnaire, which was pilot tested on six researchers, used a combination of questions and visual prompts to elicit participants’ perceptions about the bivariate relationships between 11 factors in the food and transportation system. Each participant answered questions related to a unique set of relationships within their domain of expertise.

Using CIB analysis, we identified 21 plausible future scenarios for the system. In the baseline model, ‘healthy’ scenarios (with low chronic disease, high physical activity, and low consumption of highly processed foods) were characterized by high public transportation subsidies, low car use, high street safety, and high free time, illustrating the links between transportation, free time and dietary behaviors. In analyses of interventions, low car use, high public transport subsidies and high free time were associated with the highest proportion of factors in a healthful state and with high proportions of ‘healthy’ scenarios. High political will for social change also emerged as critically important in promoting healthy systems and urban health outcomes.

The CIB method can play a novel role in augmenting understandings of complex urban systems by enabling insights into future scenarios that can be used alongside other approaches to guide urban health policy planning and action.

## 1 Introduction

Urbanization is occurring worldwide, and at an especially rapid rate in low-and-middle income countries (United Nations, 2014, 2018). Promoting urban health and health equity has become an important global focus for public health to ensure that the benefits of urbanization are shared by all. Urban health arises and is perpetuated by a system of factors spanning multiple levels and scales, including individual, social, environmental, and political factors. These factors interact through dynamic feedback processes and in non-linear ways that make their health consequences difficult to predict using simple methods (Tozan and Ompad, 2015). Coming to grips with these complexities and achieving a holistic understanding of urban health issues is precisely what is needed to enable the design of effective public health action (Diez Roux, 2011). Policies and health interventions designed without an understanding of the complex system underpinning the development and evolution of urban health issues can fall short of achieving their intended aims (Sterman, 1994).

Food and transportation systems in cities represent two facets of urban life that have widely been recognized as important determinants of health and drivers of non-communicable diseases (NCDs) (Branca et al., 2019; Litman, 2013). For instance, urban dwellers have unique access to a wide variety of fresh and processed foods sold in supermarkets, convenience stores, restaurants, and fast-food outlets. The convenience and relative availability of a variety of foods, both healthy and unhealthy, have been shown to influence dietary behavior and body weight (Barrientos-Gutierrez et al., 2017; Cobb et al., 2015). Transportation, on the other hand, can shape NCD risk through mediating factors such as physical inactivity and air pollution (Lee et al., 2012; Prüss-Ustün et al., 2019). Transportation and food systems are also interconnected: for example, transportation availability can influence ease of access to affordable healthy food, travel time (United States Department of Agriculture, 2009) and could impact the time available for food preparation and cooking. Understanding the health impacts of policies and interventions directed towards transportation and food systems also requires consideration of broader systems shaping urban health.

Several systems methods have been used to better understand the links between urban environments, urban policies, and health. With advances in computational power, researchers have increasingly pursued quantitative simulation-based methods to study the dynamic evolution of diseases at the population level. These methods include, for example, system dynamics and agent-based modelling (Carey et al., 2015; Lich et al., 2013). While the earliest applications of these methods focused on modelling infectious diseases (Willem et al., 2017), they have since been used to study obesity (Meisel et al., 2020; Morshed et al., 2019) and cardiovascular diseases (Hirsch et al., 2010), and also to understand how urban environments and urban policies impact diet (Auchincloss et al., 2011) and physical activity (Lemoine et al., 2016a, 2016b; Yang and Diez-Roux, 2013; Yang et al., 2011, 2012, 2014, 2012). The main challenge in applying simulation-based methods relates to the need for empirical data (often lacking) to parameterize and confirm the validity of the models.

In parallel to the use of quantitative methods, there has also been a proliferation of qualitative and mixed methods approaches. The utility of these approaches stems from their participatory nature and use of emic perspectives to gain insights into complex health issues. This is especially important when trying to understand complex public health issues because different stakeholders can have very different understandings of the causes and consequences of a problem and its possible solutions. Participatory methods not only allow for a more comprehensive and holistic framing of complex problems, but can also extend stakeholders’ own understanding and thereby aid in their promotion of possible solutions (Carey et al., 2015). Moreover, given that “public issues often have a common referent of experiences within the same community” (Hovmand, 2014, p.18), knowledge acquired through the engagement of local stakeholders can resonate with those who may be in a position to intervene on local issues. For example, concept mapping - a mixed methods approach also known as structured conceptualization (Trochim, 1989) - has been used to identify and prioritize a broad range of factors relevant to urban health in order to guide planning and targeted policy action (Stankov et al., 2017; Trochim and Cabrera, 2005; Velonis et al., 2018). Group model building (GMB), a participatory approach aligned with system dynamics (Hovmand, 2014), has been applied to synthesize and visually depict, using causal loop diagrams (CLDs), stakeholder’s perceptions of the relationships between urban environments and health, exploring issues such as healthy food access (Mui et al., 2019), food-related policymaking (Waqa et al., 2017), transportation (Sarmiento et al., 2020) and obesity (Savona et al., 2019). GMB has also been used to extend insights into the interrelationships between constructs identified through concept mapping and to develop formal system dynamics simulation models for policy analysis (Lich et al., 2017).

Causal loop diagrams (key products of GMB) are useful tools for conveying the structure of complex systems, including the interrelationships between key variables and feedback loops that describe the dynamics of a system (Richardson, 1999; Vennix, 1996). They can also provide indications of temporality, including effects which might transpire over relatively long time-horizons. However, CLDs are limited in their capacity to afford insight into the most likely future scenarios or states of a system. Identifying the most likely future scenarios based on the relations encoded in the CLD (in addition to information on the strength and form of these relationships) is important in identifying leverage points for intervention. Several methods have been developed to identify future scenarios; however, most do not account for mutual influences between variables in a system.

Cross-Impact Analysis methods are a family of methods that can be used to afford insights into the possible future states of systems while accounting for mutual interactions between system factors. The Cross-Impact Balance (CIB) method is a variant of these methods that provides a structured means for eliciting expert knowledge about the strength and nature of the relationships between components of a system. Importantly, it can account for the dynamic interrelationships between system factors encoded in CLDs. CIB uses this information to identify qualitative scenarios that represent the possible futures of complex systems. These future scenarios have also been described as storylines that can contextualize CLDs by illustrating a set of plausible future scenarios implied by a given CLD (Weimer-Jehle et al., 2020). These future-oriented insights can be especially important for tackling urban health issues as they can play an important role in long-term public health policy planning and action (Weimer-Jehle, 2006). However, despite its utility and application across a wide range of disciplines including climate change (Ruth et al., 2015), water systems (Schütze et al., 2019) and energy (Norouzi et al., 2020), to date there have only been three published public health applications of the CIB method (Hummel and Hoffmann, 2016; Lambe et al., 2018; Weimer-Jehle et al., 2012), and none that focus on an urban health issue.

The SALURBAL Project (*Salud Urbana en America Latina*) is a large interdisciplinary project that explores how urban environments impact environmental sustainability and health in Latin American cities (Diez Roux et al., 2019). Recognizing the importance for systems thinking in understanding and addressing complex health challenges in a rapidly urbanizing region, the SALURBAL Project convened GMB workshops to understand, using systems thinking and CLDs, the complex interrelationships between transportation and food systems and health in Latin American cities (Langellier et al., 2019). Specifically, a multidisciplinary group of local stakeholders, including food and transportation systems experts from academia, government, and nonprofit organizations as well as the private sector, participated in three regional workshops. During these workshops, stakeholders were guided through a set of structured activities that culminated in the creation of CLDs that depicted the health impact of interrelationships between factors in the food and transportation systems in Latin American (Langellier et al., 2019).

In this paper, we build on the insights gained from the SALURBAL GMB workshops by re-engaging key stakeholders using the CIB method to gain additional insight into the strength and nature of the interrelationships between food and transportation systems and their influence on health across three regions of Latin America. Using this information, we identify the most plausible future scenarios of the system which enrich our understanding of how the relations represented in the CLDs could shape the states of the system (scenarios) we are most likely to see. In addition, as a way to explore the plausible impact of interventions, we estimate how the state of each factor relates to: 1. The percentage of factors in the health promoting state across all consistent scenarios; 2. The proportion of scenarios that are healthy or unhealthy; and 3. The proportion of scenarios with low chronic disease prevalence, high physical activity, and low highly processed food consumption.

## 2 Methods

### 2.1 Expert panel

We recruited experts who attended one of the three SALURBAL GMB workshops reported on by Langellier et al. (2019). These experts, identified in consultation with SALURBAL researchers from each country, originated from countries in South and Central America and attended workshops conducted in Brazil, Peru or Guatemala. They were identified based on their expertise and working knowledge of local food and transportation systems. SALURBAL country leads sent out directed emails inviting key experts in their regions to participate, along with two follow-up emails as needed. Ultimately, a total of 16 experts spanning private, academic, non-government, and policy sectors participated in our study (Table 1).

### 2.2 Data collection

We used information elicited by a ‘dots’ activity conducted in each GMB workshop to inform the system boundary and select key factors to be incorporated in the CIB analysis. The ‘dots’ activity was one in which experts were asked to place ‘dot’ stickers next to factors in the food and transportation system they perceived to be the most important/ influential determinants of health. Once the factors were identified, we constructed a table ranking each factor based on the number of dots it received and consolidated those factors which captured the same construct under a higher-order category. From this table, we selected 11 factors from the food and transportation domain along with key health and behavioral factors that received the highest number of ‘dots’ across all three workshops (i.e., the factors that experts deemed most important). We classified these factors into three broad categories: 1) health outcome factors, including ‘prevalence of chronic disease’, ‘physical activity’, and ‘highly processed food consumption’; 2) intermediary factors (so called because they may lie in the pathway linking structural/policy factors to health outcomes), including ‘car use’, ‘free time’, and ‘street safety’ (from crime); and 3) structural/policy factors, including ‘food marketing regulations’, ‘sugar sweetened beverage/ processed food taxes’, ‘healthy food prices’, ‘public transportation subsidies’, and ‘political will for social change’. For simplicity, we restricted the focus of our CIB analysis to two possible states of each factor: ‘low’ and ‘high’.

We developed a set of instructions and an interviewer-assisted questionnaire which used a combination of questions and visual prompts, in the form of graphs, to elicit experts’ perceptions about the bivariate relationships between factors, including the strength and shape of each relationship (see Appendix A). The questionnaire was pilot tested in full on three researchers familiar with systems thinking to identify issues related to the structure of the questions and instructions provided. We refined the materials based on the feedback received and then translated the questionnaire into Spanish and Portuguese, and pilot-tested it on a further three native speakers to ensure the translations were clear and appropriately captured the meaning and intent of the questions.

Experts from each region were assigned between two and three factors within their domain of expertise, which would form the basis of the questions they were asked (about 20–30 questions per expert of a total 110 questions). In this way, each expert focused on a unique set of relationships pertaining to their area of expertise. As part of the questionnaire, experts were first asked whether a given factor had a direct influence on another factor, for example, “*Does physical activity directly influence the prevalence of chronic disease? That is, could you draw a direct arrow from physical activity to prevalence of chronic disease in a CLD?”*. If the answer was ‘no’, the expert was asked about the next pair of factors. If the answer was ‘yes,’ experts were then asked to select, from a set of options, a graph that they thought best represented the shape of this relationship. In this way, we were able to elicit information about the shape of the relationship between any given pair of factors from one expert per region. These graphs were then used to deduce the strength and direction of relationships between the states of any given pair of factors in the system on a scale of –2 ‘strongly restricting’ to +2 ‘strongly promoting’ (see linked MethodsX article for more information about the questionnaire). In so doing, we were able to explicate the interaction networks - which are embodied within three separate CIB matrices, one for each region - representing the perceived influence of food and transportation factors on health in each region from the perspective of local experts. We analyzed the interaction network/ CIB matrix of each region separately using ScenarioWizard v.4.31 (Weimer-Jehle, 2018) and present the pooled findings. We pooled the findings of the scenario analyses because our data were collected from a relatively small panel of experts from each region, which limited our ability to explore regional similarities and differences.

### 2.3 Analysis

The system explored by our paper – with 11 factors that were assigned two possible states each – had 2^11^ or 2048 unique scenarios or permutations of these factors in their various states. We first used the CIB algorithm (implemented via the ScenarioWizard v.4.31 software (Weimer-Jehle, 2018)) to perform a series of calculations to identify a subset of possible scenarios or ‘futures’ that were most consistent with experts’ judgements about the interrelationships between factors in the system (Weimer-Jehle, 2006, 2008, 2008). We refer to this set of scenarios as the baseline scenarios. For example, experts may indicate that a state of high consumption of processed food and a state of low regulation of marketing towards children both strongly promote high chronic disease prevalence. In this case, a scenario characterized by high consumption of processed food, low regulation of marketing towards children, and *high* chronic disease prevalence is more consistent and therefore plausible than a scenario with the same factor states but with *low* chronic disease prevalence. The CIB algorithm is a useful tool for sorting through the large number of possible scenarios, considering the interrelationships between all factor states, to identify those configurations of factor states that are most consistent and therefore represent the most plausible future scenarios of the system. More information about the algorithm can be found in Appendix B.

In addition to identifying the most likely baseline scenarios, we used ScenarioWizard v.4.31 (Weimer-Jehle, 2018) to explore the potential impact of an external stimulus or intervention in favor of a given factor state on the profile of consistent scenarios. We did this by fixing the state of each factor, one at a time (e.g., ‘high physical activity’ or ‘high street safety’), and then deriving the consistent scenarios under each of these conditions. The number and composition of scenarios that emerge under each of these conditions can differ substantively from the baseline scenarios. The scenarios that emerge can then be compared to those arising from the baseline scenario analysis (i.e., without external intervention) to afford insight into the relative impact of the intervention on the health-promoting profile of emerging scenarios, as well as the proportion of identified scenarios with favorable health outcomes. Specifically, we examined the potential impact of external interventions from three different perspectives, each affording increasingly granular insight into the system. The three perspectives we adopted focused on: 1. the system as a whole; 2. the scenario categories (which, for the most part, were defined based on the state of all three health outcome variables combined), and 3. each of the three health outcomes individually. To understand the impact of interventions at the system-level, we explored the association between the state of each individual factor and the percentage of factors in the health-promoting state across all consistent scenarios. We then examined the proportion of scenarios that emerged across four different categories. These included ‘healthy’, ‘mixed’ or ‘unhealthy’ scenarios, which were defined based on the health promoting or health restricting state of all three health outcome variables, namely, chronic disease prevalence, physical activity, and highly processed food consumption. Scenarios categorized as ‘healthy’ featured all three health outcome variables in a health promoting state (i.e., low chronic disease prevalence, high physical activity and low processed food consumption). ‘Unhealthy’ scenarios, on the other hand, featured all three health outcomes in a health restricting state, while scenarios in the ‘mixed’ category included health outcomes in both health promoting and health restricting states. The ‘salubrious system’ scenarios, a subset of the ‘healthy’ and the ‘mixed’ scenarios, were defined as those with eight or more factors in a healthy or health promoting state. Finally, we explored how the state of each individual factor affected the proportion of consistent scenarios with each healthy outcome (i.e., low chronic disease prevalence, high physical activity and low processed food consumption).

The study protocol was reviewed by the Drexel University Institutional Review Board, which determined that the study did not constitute research involving human subjects (IRB ID: 1709005674).

## 3 Results

Of the 110 possible relationships between the 11 system factors, the highest number of bivariate relationships were identified by respondents in Guatemala (n = 60), closely followed by Peru (n = 53), with the fewest elicited in Brazil (n = 39). A large proportion of these relationships were non-linear, including 87% of all identified relationships in Brazil, while in Peru and Guatemala, experts respectively identified 81% and 77% of all relationships as non-linear.

### 3.1 Baseline scenarios

**Fig. 1:**
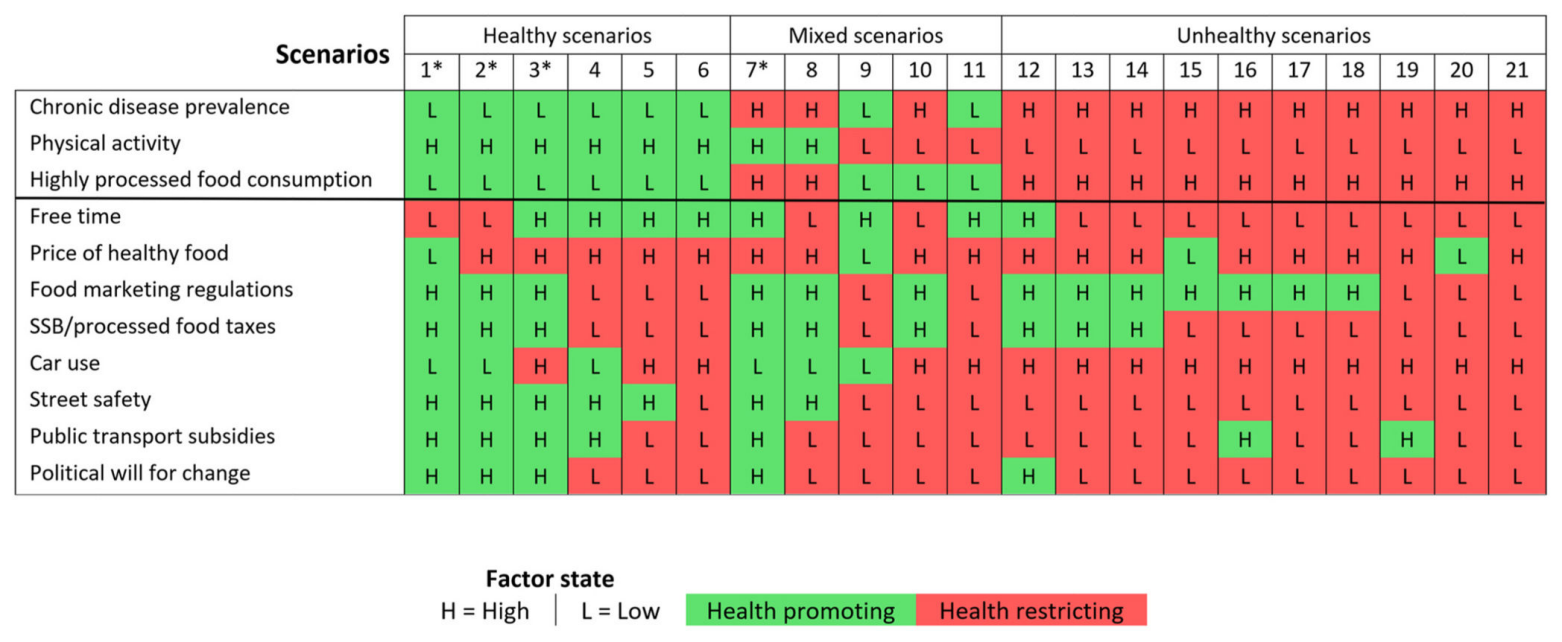
Baseline scenario table showing the consistent scenarios or possible ‘futures’ identified using the CIB analysis for the three regions. These scenarios are represented in the numbered columns while the rows identify the key factors in the system. The cells in the table characterize the state of each factor within the system, where ‘L’ refers to a ‘low’ state and ‘H’ refers to a ‘high’ state. The colors highlight whether a given factor is in a health promoting (green) or health restricting (red) state while the higher-order column categories characterize whether scenarios were considered ‘healthy’, ‘mixed’ or ‘unhealthy’ based on the health promoting or health restricting state of the three outcome variables, namely, chronic disease prevalence, physical activity, and highly processed food consumption. Scenarios with an asterisk (*) are ‘salubrious system’ scenarios, defined as those with eight or more factors (>70%) in a healthy or health promoting state. (For interpretation of the references to color in this figure legend, the reader is referred to the Web version of this article.)

The outcomes of the baseline scenario analyses are shown in Fig. 1. A total of 21 scenarios were identified across the three regions, of which 19 scenarios were unique. Based on the health promoting or health restricting state of the factors, the 21 identified scenarios were classified into four categories: ‘healthy’ (n = 6), ‘mixed’ (n = 5), ‘unhealthy’ (n = 10), and ‘salubrious system’ (n = 4; representing a subset of the ‘healthy’ and ‘mixed’ scenarios and identified by an asterisk in Fig. 1).

The six baseline scenarios classified as ‘healthy’ were characterized by low chronic disease prevalence, high physical activity, and low consumption of highly processed food. Of these healthy scenarios, the majority were also uniquely characterized by high street safety (five of six scenarios), high healthy food prices (five of six scenarios), free time (four of six scenarios), and public transportation subsidies (four of six scenarios).

In contrast, the 10 baseline scenarios classified as ‘unhealthy’ featured high chronic disease prevalence, low physical activity and high consumption of highly processed foods. These unhealthy scenarios were also characterized by high car use and low street safety (all scenarios), as well as low political will for social change (nine of ten scenarios), low free time (nine of ten scenarios), and low public transportation subsidies and high health food prices (eight of ten scenarios each).

Of the five ‘mixed’ health outcome scenarios, two had high chronic disease prevalence and high consumption of processed foods but high physical activity; two had low chronic disease prevalence and low consumption of processed foods but low physical activity; and one had high chronic disease prevalence and low physical activity but low consumption of highly processed foods. Of these five ‘mixed’ scenarios, the majority were also characterized by low political will for social change and low public transportation subsidies (four of five scenarios each) as well as high healthy food prices (four of five scenarios).

We also identified four ‘salubrious system’ scenarios (a subset of the ‘healthy’ and ‘mixed’ outcome scenarios) that were characterized by the presence of eight or more factors (>70%) in a healthy or health promoting state. Moreover, these ‘salubrious system’ scenarios were all characterized by high physical activity, high street safety and health promoting structural/policy factors including high political will for social change, high food marketing regulations, high sugar-sweetened beverage and processed food taxes and high public transportation subsidies. Most of these scenarios (three of four) were also characterized as having low chronic disease prevalence, low consumption of highly processed food and low car use.

### 3.2 Consistent scenarios associated with external interventions

To gain insight into the potential impact of external interventions on the system, we examined how the state of each individual factor affects: 1. the percentage of factors in the health promoting state across all consistent scenarios; 2. the proportion of scenarios of each category (‘salubrious system’, ‘healthy’, ‘mixed’ and ‘unhealthy’) among all consistent scenarios; and 3. the proportion of consistent scenarios with each healthy outcome (low chronic disease prevalence, high physical activity, and low consumption of highly processed food). This allowed us to assess the set of system ‘futures’ that may plausibly eventuate from fixing each factor in a specific state (e.g., enacting high public transport subsidies). Importantly, the frequencies presented as part of this analysis do not indicate probabilities and therefore should not be read with a probabilistic interpretation.

#### 3.2.1 Impacts at the systems-level

**Fig. 2:**
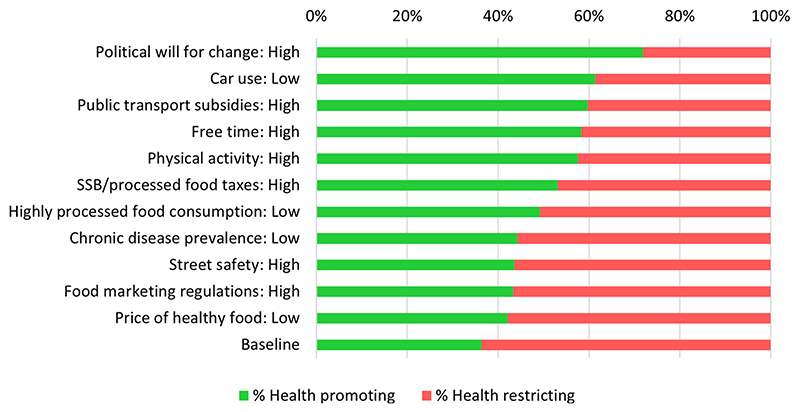
The percentage of factors in a health promoting state (green) across all identified consistent scenarios at baseline and when fixing or stabilizing the state of a given factor (y-axis). (For interpretation of the references to color in this figure legend, the reader is referred to the Web version of this article.)

At the systems level, the highest proportion of factors in health promoting states emerged from scenarios where political will for social change was fixed to high (72% factors in health promoting states), followed by those with low car use (61% factors in health promoting states) and scenarios with public transportation subsidies in a ‘high’ state (60% factors in health promoting states) (Fig. 2).

#### 3.2.2 Impacts on scenario categories

**Fig. 3:**
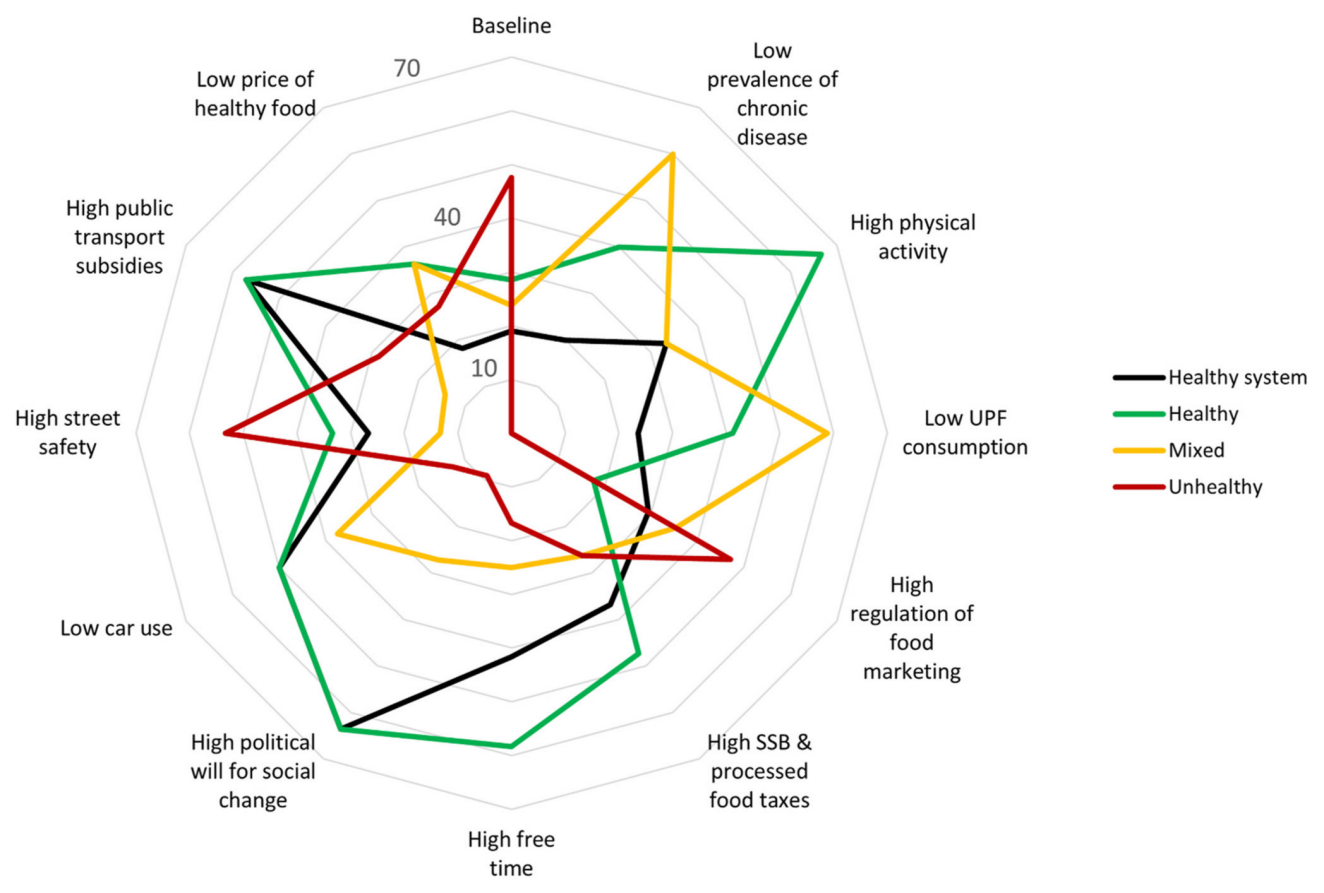
Radar plot showing the baseline scenario and the impact of fixing or stabilizing a given factor state on the percentage of consistent scenarios defined as ‘healthy’ (green), ‘mixed’ (yellow), ‘unhealthy’ (red) and ‘salubrious system’ (black) scenarios. ‘Healthy’ outcome scenarios were those with low chronic disease prevalence, high physical activity, and low consumption of highly processed food, while ‘unhealthy’ scenarios were those with high chronic disease prevalence, low physical activity, and high consumption of highly processed food. ‘Mixed’ scenarios included chronic disease prevalence, physical activity and highly processed food consumption outcomes that were in both health promoting and health restricting states. The ‘salubrious system’ scenarios (a subset of the ‘healthy’ and ‘mixed’ outcome scenarios) were characterized by eight or more (i.e., >70%) factors in a health promoting state. (For interpretation of the references to color in this figure legend, the reader is referred to the Web version of this article.)

High political will for social change and high public transportation subsidies were each associated with high proportions (over half) of scenarios in the ‘salubrious system’ or ‘healthy’ outcome categories. Fixing free time in a high state was also associated with a high proportion of scenarios in the ‘healthy’ outcome category (Fig. 3). Moreover, across all the external intervention scenarios (Appendix C), the majority of ‘healthy’ scenarios that emerged included high street safety and low car use.

Some proportion of ‘unhealthy’ scenarios was observed for all the states tested, but the lowest proportion of ‘unhealthy’ outcome scenarios was observed for high levels of political will for social change (Fig. 3). All ‘unhealthy’ outcome scenarios, arising across all external interventions, also included high car use and low street safety (Appendix C). No clear pattern was observed for ‘mixed’ outcome scenarios, as no one factor was associated with more than half the ‘mixed’ outcome scenarios.

#### 3.2.3 Impacts on individual health outcomes

**Fig. 4:**
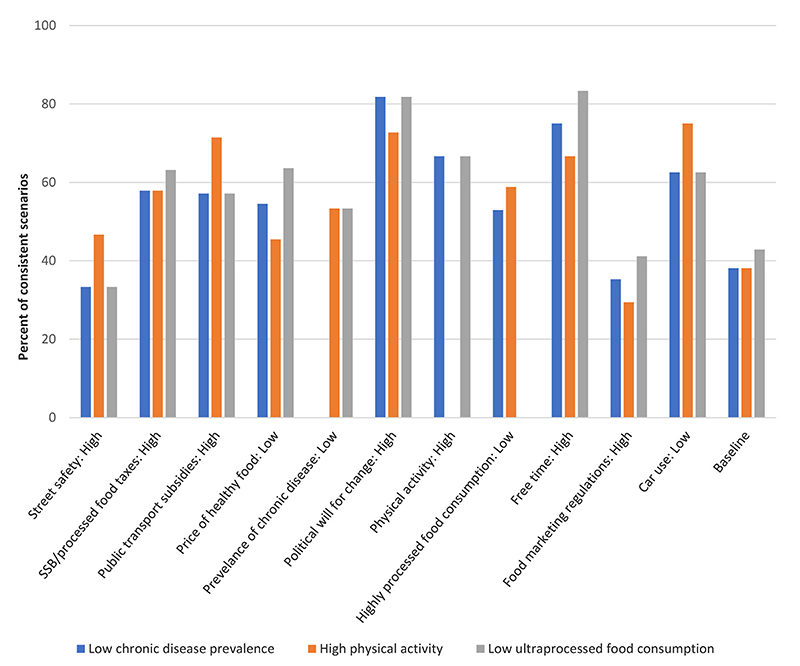
Percentage of consistent scenarios with low chronic disease prevalence (blue), high physical activity (orange) and low consumption of highly processed food (gray) at baseline and those that arise from the stabilizing influence of a given factor state (x-axis) across the three regions. (For interpretation of the references to color in this figure legend, the reader is referred to the Web version of this article.)

As shown in Fig. 4, high political will for social change was associated with a high percentage of scenarios with low chronic disease prevalence, high physical activity, and low consumption of highly processed food. High free time and low car use were also associated with a high percentage of scenarios with low chronic disease prevalence and low consumption of highly processed foods, while low car use and high public transportation subsides were associated with high physical activity.

## 4 Discussion

We used the CIB method to strengthen our understanding of how food and transportation systems impact health in Latin America. Unlike other systems approaches, the CIB method enabled us to uniquely characterize the strength and nature of the interrelationships between 11 factors in the food and transportation system. This information augments the insights gained from the CLDs. For instance, we found that most relationships between system factors were perceived to be nonlinear, which can either exponentially amplify or dampen policy impacts. Moreover, our application of the CIB method afforded unique insight into the most plausible future scenarios, or combinations of factor states, in the region – both in the presence and absence of external intervention. The storylines that emerged through the identification of these scenarios provide important contextual information that can be leveraged to guide policy planning and action. For example, using the baseline scenario analysis, we identified 21 consistent scenarios across the three regions, of which several were ‘healthy’ scenarios. We further used the CIB analysis to examine how the state of each system-level factor was associated with the other factors to gain insights into the potential impact of external interventions on the system. Through this process, we identified factors such as political will for social change and public transportation subsidies that were associated not only with scenarios characterized by positive health outcome states, but also health promoting storylines.

We identified six ‘healthy’ scenarios which were defined by low chronic disease prevalence, low processed food consumption, and high physical activity. The main difference between these scenarios and the 10 scenarios categorized as ‘unhealthy’ was that the ‘healthy’ outcome scenarios were characterized by storylines that featured high public transportation subsidies, low car use, high street safety and free time. The relation between car use and higher chronic disease risk has been demonstrated by several studies. For example, car use has been associated with higher body mass index (Dons et al., 2018) and less physical activity (Morckel and Terzano, 2014). Higher perceived safety has also been linked to lower chronic disease risk, including more leisure-time physical activity (Arango et al., 2013) and lower odds of stroke, cancer, and obesity among residents of low crime neighborhoods (Freedman et al., 2011; Richardson et al., 2017). A novel finding of our CIB scenario analysis however, was that a constellation of factors, including low car use and high safety from crime, as well as high public transport subsidies and high free time were related to a range of positive health outcomes including not only lower chronic disease prevalence and higher physical activity, but also lower consumption of highly processed foods. This finding highlights the possible interconnection between transportation systems and dietary behavior, possibly mediated by the free time available for food purchasing and cooking.

In exploratory analyses of external interventions, low car use, high public transportation subsidies and high free time were also associated with the highest proportion of factors in a healthful state (across all scenarios). They were also associated with high proportions of healthy scenarios (slightly less so for low car use). These results reinforce the key role of these factors in shaping health in the cities of Latin America as perceived by the stakeholders engaged in our study. The interacting effects of transportation and free time in shaping both physical activity and diet were also highlighted in the GMB workshops (Langellier et al., 2019). Specifically, the CLDs identify how public and active transportation use can increase time spent engaging in physical activity both directly and indirectly by decreasing car use and congestion and enabling free time for such activity. More free time also increases opportunities for cooking at home, which reduces the consumption of highly processed foods. Over time, increases in physical activity and reduced consumption of unhealthy foods represent important health-promoting influences in the CLD. The interplay of these factors has not been evaluated in the Latin American context, where most studies of the transportation environment are conducted separately from those in the food environment.

Several empirical studies support the insights that emerged from the scenario analysis, particularly the influence of car use and free time on the consumption of ultra-processed foods and chronic disease outcomes, and of car use and public transportation subsidies on physical activity. For example, a large cross-sectional study of Australian adults found that longer driving times were associated with obesity (Ding et al., 2014), while another study based in Norway showed that parents with medium and high time scarcity were at much higher odds of consuming ultra-processed foods compared to parents with low time scarcity (Djupegot et al., 2017). Similar patterns have been observed among US adults (Horning et al., 2017). In one study, focus group participants explained how their decision to purchase unhealthy foods was influenced by their moods and that they were much more likely to buy ultra-processed foods during shopping trips when they were pressed for time or hungry (Moran et al., 2019). There is also evidence that public transportation subsidies have the capacity to induce changes in travel patterns, including shifts from car to public transportation use, and increases in physical activity among older adults (Webb et al., 2016).

Another notable finding was the perceived influence of political will for social change. In the baseline scenario analysis, the unhealthy scenarios were significantly more likely to include low political will for social change. In contrast, high political will for social change was associated with ‘salubrious system’ scenarios which were characterized by low chronic disease prevalence, high physical activity, and low consumption of highly processed foods. In the analysis of external interventions, high political will for social change was associated with the highest proportion of ‘salubrious system’ and ‘healthy’ outcome scenarios, and the lowest proportion of ‘unhealthy’ outcome scenarios. It was also associated with the highest percentage of other factors in a health promoting state. The critical role of political will for social change in the urban systems driving health is an important insight from these analyses.

As demonstrated by our study, the CIB method can strengthen public health inquiry by augmenting insights into complex systems. It has the capacity to explore the entire space of possible future scenarios of a system and to identify those that are most plausible, both in the presence and absence of external intervention (Weimer-Jehle et al., 2020). The storylines that emerge through the identification of these scenarios can provide important contextual information to guide policy planning and action. For example, the finding that ‘healthy’ outcome scenarios all feature low car use and high street safety signals the potential salience of these factors in the urban system and highlights the importance of developing policies with these factors in mind. By characterizing scenarios compatible with a set of relationships between factors, the CIB approach allows a more holistic understanding and characterization of the systems that drive health and of the factors that might be most influential in nudging the system in a specific direction. A further strength of the CIB approach is its capacity to engage experts not necessarily familiar with complex systems modelling, but with knowledge directly relevant to urban health. As such, the outcomes that emerge can facilitate interdisciplinary discourse and promote a shared understanding of the possible future scenarios for cities. Understanding these scenarios can contextualize and, in some respects, sensitize and inform further qualitative and simulation-based inquiry. For example, the CIB method and its findings can contribute information about the strength and nature of relationships between sets/ ensembles of factors in the system which can seed discussions in prospective GMB workshops. CIB can complement information gained from empirical studies and other methods to inform the specification of key parameters and the shape of the relationships between them in quantitative models, such as system dynamics and agent-based models. Insights gained from CIB can also provide intuitions about the relative effectiveness of interventions which could be tested and explored more formally using simulation-based platforms, including agent-based or system dynamics models.

There are also notable challenges to applying CIB analysis. The time commitment and burden on experts is commonly cited as a barrier to the use of CIB analysis across a range of disciplines (Weimer-Jehle et al., 2020). This time burden arises because CIB requires experts to make judgements about the possible interactions between every pair of factors in the system, and all their states. To alleviate this burden, we limited the scope of our analysis to include a set of 11 factors that we perceived to be the most important and influential by stakeholders involved in the GMB workshops. This prioritization meant that the scenarios that emerged through our analysis represented relatively aggregate descriptions of the system, instead of the types of nuanced and detailed storylines that could have emerged from an analysis including a larger number of factors. This also limits the generalizability of the insights. As such, our findings ought to be interpreted qualitatively and alongside other representations of systems, such as CLDs and simulation-based models, particularly when it comes to policy planning. It may also be possible that the way we translated and encoded the graph selections of experts into the CIB matrix did not truly capture their conception of the relationships between factor states in the system. Another limitation concerning CIB relates to the potential for specification error (Weimer-Jehle et al., 2020). That is, since the information collected about the interactions between factors in the system represent the worldviews of the experts making the judgments, we may have inadvertently omitted experts with important insights that could bias the storylines that emerged from our analyses. This concern is especially important in CIB where there is no easy way to quantitatively reconcile differing judgments about the same set of relationships across different experts. For this reason, we used a relatively small sample of experts from each region and collected information about each relationship from only one expert per region. This limited our ability to explore regional similarities and differences, as any observed variation could have arisen from personal differences in experts’ perceptions rather than true regional differences. While we ultimately decided to pool the results of the three CIB analyses, it is important to note that these analyses can also present an opportunity to explore the most striking differences between regional scenarios. For example, stark differences between the most internally consistent scenarios from each region could signify regional variation in experts’ views of the system. The relationships underpinning these differences could then be traced in the interaction network encoded in the CIB matrix and further interpreted. An important area for future research concerns advancing this method to enable consensus-building across differing quantitative judgements elicited from different experts.

## 5 Conclusion

The CIB method is useful as a systems thinking approach capable of advancing insights into urban health issues. This method allows researchers to elicit information concerning the perceived strength and nature of relationships between key system factors, including important non-linearities between components of the system. Moreover, scenario analyses can contribute valuable qualitative insights into the possible futures of a system. For example, our findings emphasize the importance of high political will for social change given its association with scenarios characterized by positive health outcome states and health promoting storylines. Additionally, our findings suggest that low car use and high street safety from crime could play an important role in promoting ‘salubrious system’ and ‘healthy’ outcome scenarios in cities across the region. Moreover, we found that interventions promoting low car use, high public transportation subsidies, and more free time were associated with future scenarios characterized by favorable health outcomes, including low chronic disease prevalence, high physical activity, and low processed food consumption. The insights gained from our study align with several relationships identified in the CLDs presented by Langellier et al. (2019) and can be used to contextualize these using future-oriented storylines pertinent to the Latin American context. Our analyses may additionally provide intuitions about potentially effective policies and interventions that could correspond with systems that are more supportive of health and wellbeing.

## Supplementary Material

Appendix A. Supplementary data

Supplementary data to this article can be found online at https://doi.org/10.1016/j.socscimed.2021.114157.

Multimedia component 1

Multimedia component 2

Multimedia component 3

## Figures and Tables

**Table 1:** Characteristics of experts involved in the cross-impact balance analysis.

		South America (N = 11)	Central America (N = 5)
Characteristics			
GMB workshop region attended	São Paulo, Brazil (5)	Lima, Peru (6)	Antigua, Guatemala (5)
Country	Brazil (5)	Peru (4) Argentina (2)	Guatemala (3) Méexico (1) Panamé (1)
Expertise	Food systems (2), Transportation (3)	Food systems (3), Transportation (3)	Food systems (3), Transportation (2)
Gender	Female (2), Male (3)	Female (4), Male (2)	Female (2), Male (3)
Stakeholder type	Private sector (1), Academic (1), NGO (2), Policymaker (1)	Academic (2), NGO (2), Policymaker (2)	Academic (2), NGO (2), Policymaker (1)
Highest level of education	High school (1), Doctorate (4)	Bachelor (2), Masters (3), Doctorate (1)	Bachelor (1), Masters (4)

GMB = group model building; NGO = non-government organization.
