# Supplementary material for: From causal loop diagrams to future scenarios: Using the cross-impact balance method to augment understanding of urban health in Latin America
Source: Soc Sci Med. Author manuscript; Available in PMC 2021 Aug 1. (PMC8287591; doi:10.1016/j.socscimed.2021.114157)
Supplement: Multimedia component 1 [file EMS131094-supplement-Multimedia_component_1.docx]

# Appendix A: CIB Questionnaire and matrix

Participants are first asked a stem question of the form “Does factor x directly influence factor y?”. An example from our Portuguese questionnaire is provided below. The question asks if physical actiivty has a direct influence on the prevalence of chronic disease. Participants must select ‘yes’ or ‘no’. If they answer ‘no’, then they are asked about the relationship between physical activity and the next factor.


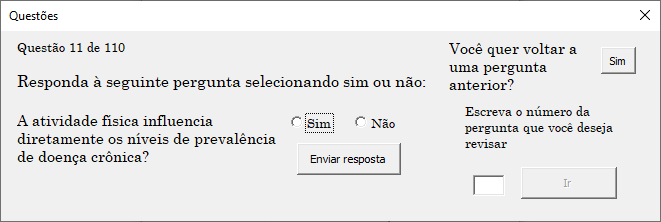


If they answer ‘yes’, that is, they think that physical activity directly influences chronic disease prevalence, then they are presented with a set of linear and non-linear graphs (shown below in Figure A1) and asked to select the graph that best describes the relationship between physical activity and chronic disease prevalence, and where applicable, to select the line that best describes the strength of the relationship (i.e., the solid line (1) or the dashed line (2)).


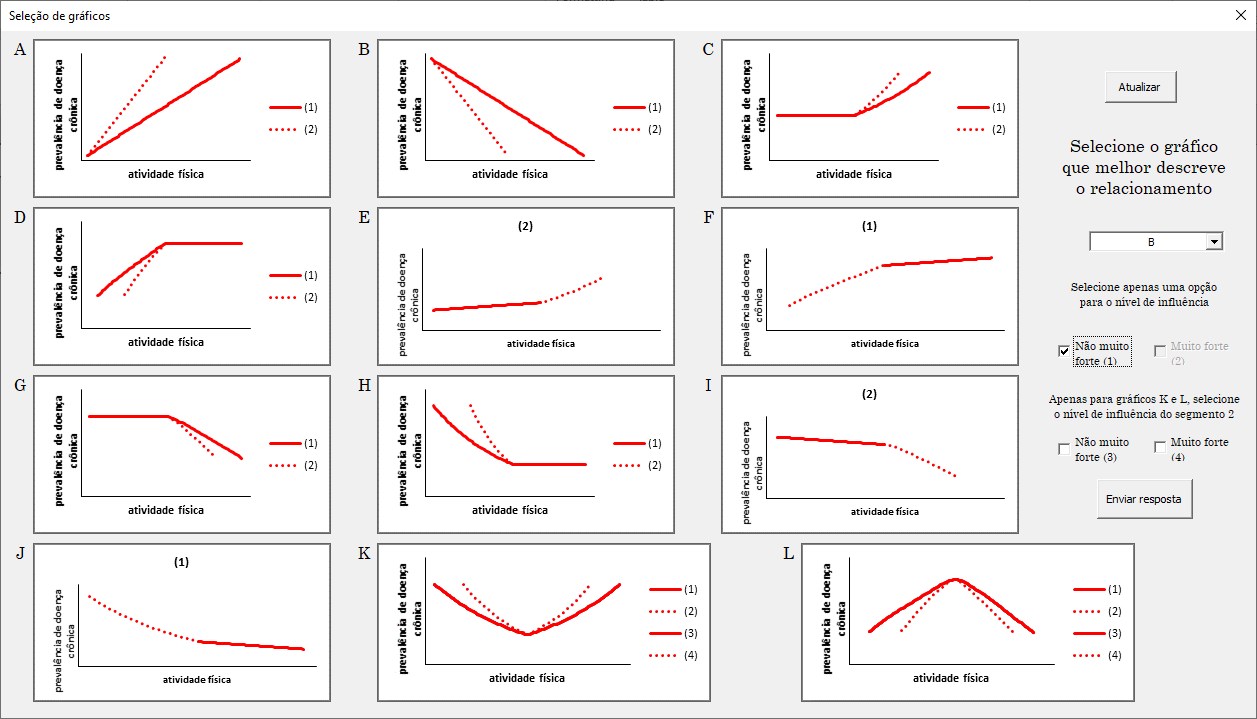


**Figure A1:** List of all possible bivariate relationships from which participants selected the one they perceived best describes the influence of one factor on another.

Each selection from Figure A1 is then translated into the CIB matrix in the form of a numeric code (listed in Table A1, below) which represents the strength and direction of the selected relationship on a scale of -2 to +2. Where the numbers on the scale represent:

+2: strongly promoting direct influence
+1: weakly promoting direct influence
0: no direct influence
-1: weakly restricting direct influence
-2: strongly restricting direct influence

| **Table A1:** Graph selection & corresponding values in the CIB matrix | | | | | |
| --- | --- | --- | --- | --- | --- |
|  |  | **CIB MATRIX** | **Target of influence  (y-axis factor)** | | |
| **Graph selection**  graph(line) |  | **Source of influence** |  | **H** | **L** |
| A(1) |  | **x-axis factor** | **H** | 1 | -1 |
|  |  |  | **L** | -1 | 1 |
| A(2) |  | **x-axis factor** | **H** | 2 | -2 |
|  |  |  | **L** | -2 | 2 |
| B(1) |  | **x-axis factor** | **H** | -1 | 1 |
|  |  |  | **L** | 1 | -1 |
| B(2) |  | **x-axis factor** | **H** | -2 | 2 |
|  |  |  | **L** | 2 | -2 |
| C(1) |  | **x-axis factor** | **H** | 1 | -1 |
|  |  |  | **L** | 0 | 0 |
| C(2) |  | **x-axis factor** | **H** | 2 | -2 |
|  |  |  | **L** | 0 | 0 |
| D(1) |  | **x-axis factor** | **H** | 0 | 0 |
|  |  |  | **L** | -1 | 1 |
| D(2) |  | **x-axis factor** | **H** | 0 | 0 |
|  |  |  | **L** | -2 | 2 |
| E(2) |  | **x-axis factor** | **H** | 2 | -2 |
|  |  |  | **L** | -1 | 1 |
| F(1) |  | **x-axis factor** | **H** | 1 | -1 |
|  |  |  | **L** | -2 | 2 |
| G(1) |  | **x-axis factor** | **H** | -1 | 1 |
|  |  |  | **L** | 0 | 0 |
| G(2) |  | **x-axis factor** | **H** | -2 | 2 |
|  |  |  | **L** | 0 | 0 |
| H(1) |  | **x-axis factor** | **H** | 0 | 0 |
|  |  |  | **L** | 1 | -1 |
| H(2) |  | **x-axis factor** | **H** | 0 | 0 |
|  |  |  | **L** | 2 | -2 |
| I(2) |  | **x-axis factor** | **H** | -2 | 2 |
|  |  |  | **L** | 1 | -1 |
| J(1) |  | **x-axis factor** | **H** | -1 | 1 |
|  |  |  | **L** | 2 | -2 |
| K(1,3) |  | **x-axis factor** | **H** | 1 | -1 |
|  |  |  | **L** | 1 | -1 |
| K(1,4) |  | **x-axis factor** | **H** | 2 | -2 |
|  |  |  | **L** | 1 | -1 |
| K(2,3) |  | **x-axis factor** | **H** | 1 | -1 |
|  |  |  | **L** | 2 | -2 |
| K(2,4) |  | **x-axis factor** | **H** | 2 | -2 |
|  |  |  | **L** | 2 | -2 |
| L(1,3) |  | **x-axis factor** | **H** | -1 | 1 |
|  |  |  | **L** | -2 | 1 |
| L(1,4) |  | **x-axis factor** | **H** | -2 | 2 |
|  |  |  | **L** | -1 | 1 |
| L(2,3) |  | **x-axis factor** | **H** | -1 | 1 |
|  |  |  | **L** | -2 | 2 |
| L(2,4) |  | **x-axis factor** | **H** | -2 | 2 |
|  |  |  | **L** | -2 | 2 |

H = High; L = Low

Below we provide three examples of how the graphs selected by participants from Peru were interpreted and translated into a numeric code featured in the CIB matrix.

| **Graph selection** | **Interpretation** | **Values in CIB matrix** |
| --- | --- | --- |
| 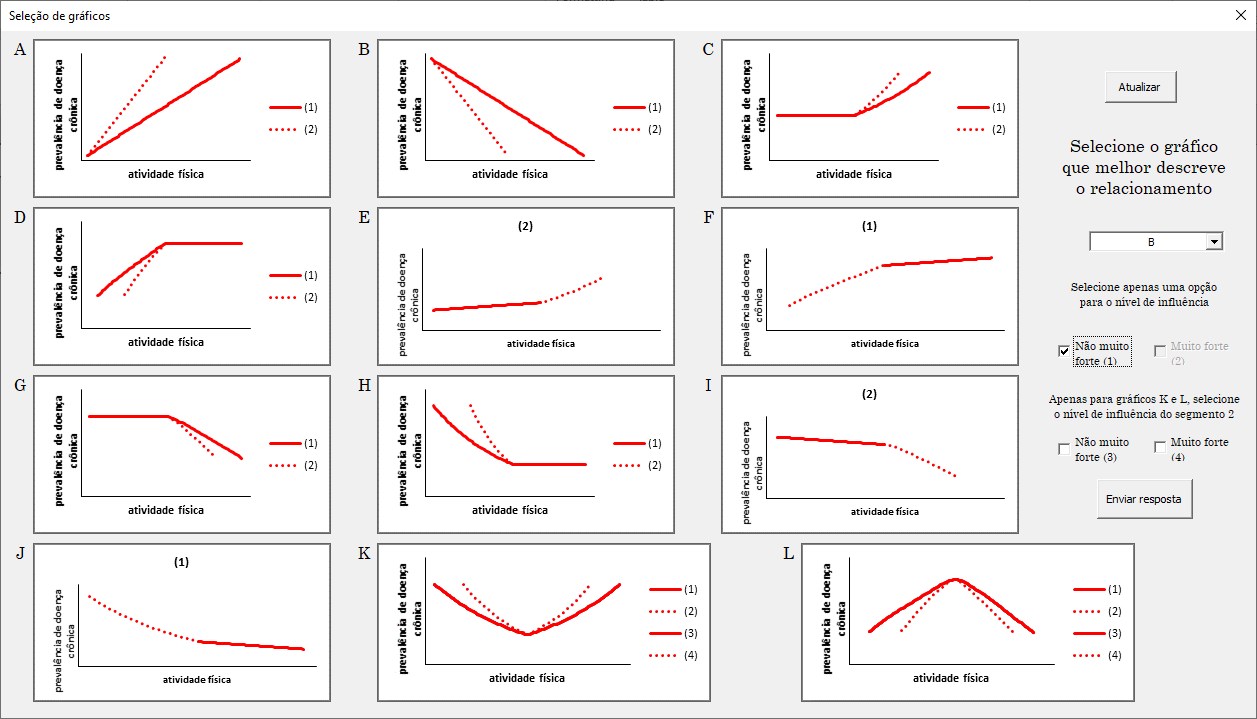  **Prevalence of chronic disease**  **Physical activity** | At high levels of physical activity, more physical activity weakly:  >> restricts the high chronic disease state  >> promotes the low chronic disease state  At low levels of physical activity, lower physical activity strongly:  >> restricts low chronic disease prevalence >> promotes high chronic disease prevalence | \| *its corresponding numeric value in the matrix*  *The influence is considered weakly restricting because at high levels of physical activity, the line slopes gently. As such, a ‘-1’ is entered in the high physical activity & high chronic disease cell. At low levels, the slope of the line is much steeper so a ‘-2’ is entered in the low chronic disease cell. The colours link each statement to* \| \| **Prevalence of chronic disease** \| \| \| --- \| --- \| --- \| --- \| \| **High** \| **Low** \| \| \| **B. Physical activity** \| **High** \| **-1** \| **1** \| \| **Low** \| **2** \| **-2** \| |
| 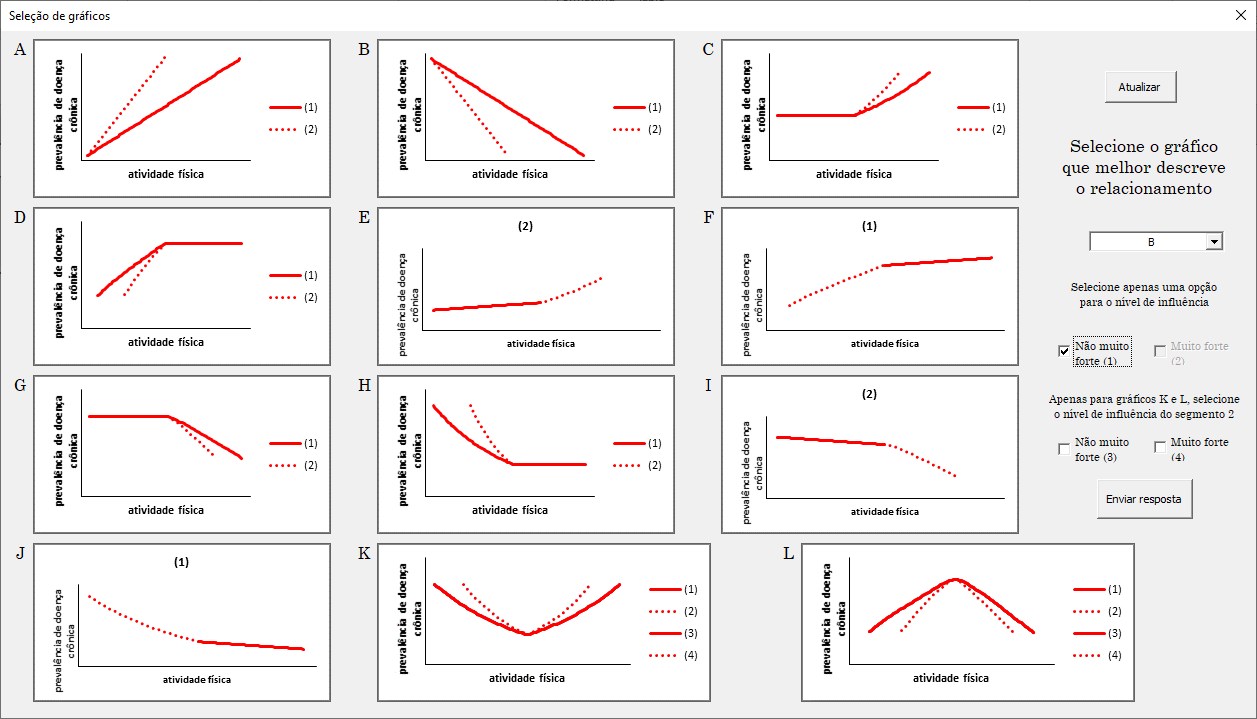  **Consumption of highly processed food**  **Prevalence of chronic disease** | At high levels of UPF consumption, higher UPF consumption weakly:  >> promotes the high chronic disease state  >> restricts the low chronic disease state  At low levels of UPF consumption, lower UPF consumption strongly: >> promotes low chronic disease prevalence  >> restricts high chronic disease prevalence | \|  \| \| **Prevalence of chronic disease** \| \| \| --- \| --- \| --- \| --- \| \| **High** \| **Low** \| \| \| **C. Consumption of highly processed food** \| **High** \| **2** \| **-2** \| \| **Low** \| **-2** \| **2** \| |
| **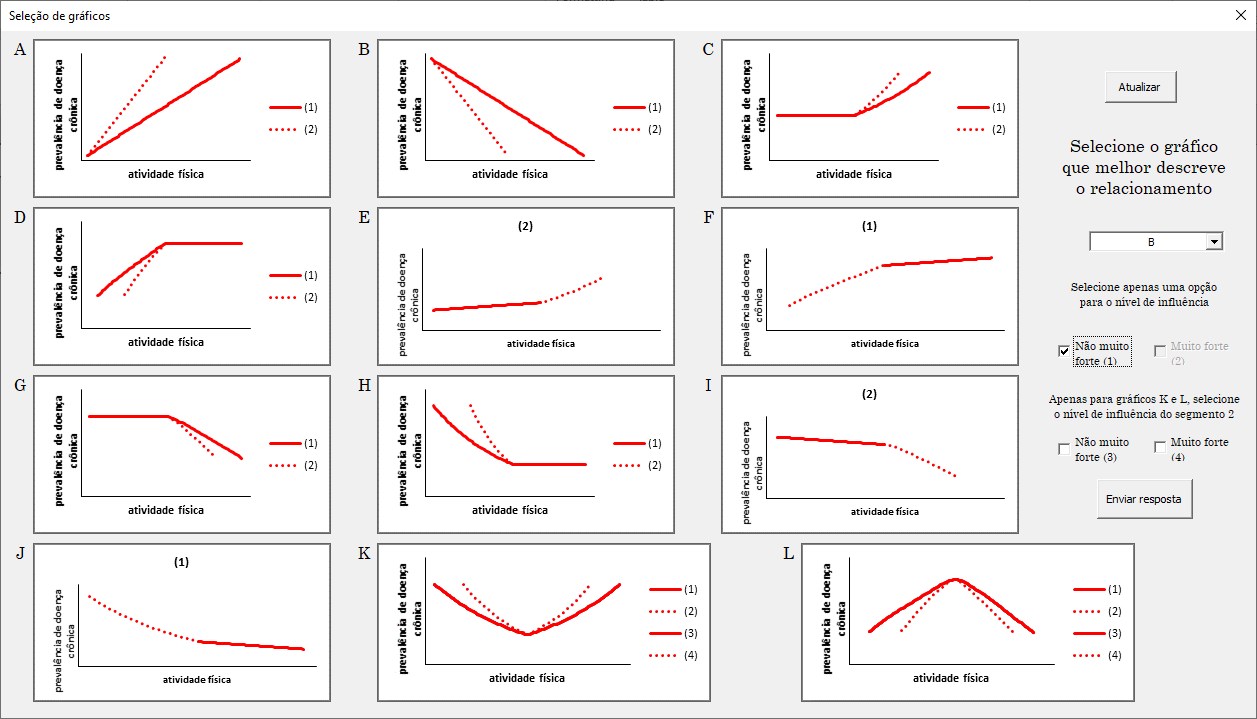**  **Prevalence of chronic disease**  **SSB/ processed food taxes** | At high levels of sugar sweetened beverage (SSB)/ processed food taxes, higher taxes weakly: >> promote the low chronic disease state  >> restrict the high chronic disease state  At low levels of SSB/ processed food taxes, lower taxes have no influence on chronic disease prevalence. | \|  \| \| **Prevalence of chronic disease** \| \| \| --- \| --- \| --- \| --- \| \| **High** \| **Low** \| \| \| **E. Sugar sweetened beverage/ processed food tax** \| **High** \| **-1** \| **1** \| \| **Low** \| **0** \| **0** \| |
