# Supplementary material for: From causal loop diagrams to future scenarios: Using the cross-impact balance method to augment understanding of urban health in Latin America
Source: Soc Sci Med. Author manuscript; Available in PMC 2021 Aug 1. (PMC8287591; doi:10.1016/j.socscimed.2021.114157)
Supplement: Multimedia component 2 [file EMS131094-supplement-Multimedia_component_2.docx]

# Appendix B: CIB algorithm & the identification of consistent scenarios

The ScenarioWizard software uses an algorithm that identifies consistent scenarios by enumerating all possible scenarios and then checking each one in turn to determine if it contains any contradictions. Given that in this study the CIB matrix has 11 factors with two possible states each, there exist 2^11^= 2048 possible combinations by which these factors and their levels can be arranged into unique scenarios. However, only scenarios with one or no contradictions are accepted as consistent. Below we use the CIB matrix for Peru to demonstrate how the CIB algorithm works. The first step involves marking the rows that define a scenario; in our example we use blue shading to characterize the factor states that comprise the example scenarios (i.e., low chronic disease prevalence, high physical activity, high consumption of processed food, high regulation of food marketing towards children, high sugar-sweetened beverage/ processed food taxes, high free time, low car use, high street safety, high public transportation subsidies and low price of healthy food). Second, the influence of each factor state in the scenario on every other factor is evaluated. This is done by combining or ‘balancing’ all the influences on a given factor (of which there can be several). For instance, in the example scenario, the prevalence of chronic disease (in the first column) is influenced by all factor states represented in the rows of the matrix, except for ‘low car use’ and ‘price of healthy food’ (both with zeros in all four cells of the first column). To account for all these influences on a given column descriptor state (i.e., high or low), the CIB algorithm sums the values in the blue cells (example scenario) and records the value in the impact balance row at the bottom of the matrix (e.g., high chronic disease prevalence: -1+2-1-1-1-1+0-1-2+0=-6).

| **PERU  CIB matrix** | | **Prevalence of chronic disease** | | **Physical activity** | | **Consumption of highly processed food** | | **Regulation concerning food marketing toward children** | | **Sugar-sweetened beverage/ processed food tax** | | | **Amount of free time** | | | **Political will for social change** | | | **Car use** | | | **Street safety** | | **Public transport subsidies** | | **Price of healthy food** | |
| --- | --- | --- | --- | --- | --- | --- | --- | --- | --- | --- | --- | --- | --- | --- | --- | --- | --- | --- | --- | --- | --- | --- | --- | --- | --- | --- | --- |
|  |  | **High** | **Low** | **High** | **Low** | **High** | **Low** | **High** | **Low** | **High** | **Low** | **High** | | **Low** | **High** | | **Low** | **High** | | **Low** | **High** | | **Low** | **High** | **Low** | **High** | **Low** |
|  |  |  |  |  |  |  |  |  |  |  |  |  |  |  |  |  |  |  |  |  |  |  |  |  |  |  |  |
| **A. Prevalence of chronic disease** | **High** |  |  | 0 | 0 | 0 | 0 | 2 | -2 | 2 | -2 | 0 | | 0 | 1 | | -1 | 0 | | 0 | 0 | | 0 | 0 | 0 | 2 | -2 |
|  | **Low** |  |  | 0 | 0 | 0 | 0 | -1 | 1 | -1 | 1 | 0 | | 0 | 1 | | -1 | 0 | | 0 | 0 | | 0 | 0 | 0 | -2 | 2 |
| **B. Physical activity** | **High** | -1 | 1 |  |  | 0 | 0 | 1 | -1 | 1 | -1 | -2 | | 2 | 1 | | -1 | -2 | | 2 | 2 | | -2 |  |  | 1 | -1 |
|  | **Low** | 2 | -2 |  |  | 0 | 0 | 0 | 0 | 0 | 0 | 0 | | 0 | -2 | | 2 | 1 | | -1 | -1 | | 1 |  |  | 0 | 0 |
| **C. Consumption of highly processed food** | **High** | 2 | -2 | 0 | 0 |  |  | 0 | 0 | 0 | 0 | 0 | | 0 | 0 | | 0 | 0 | | 0 | 0 | | 0 | 0 | 0 | 1 | -1 |
|  | **Low** | -2 | 2 | 0 | 0 |  |  | 0 | 0 | 0 | 0 | 0 | | 0 | 0 | | 0 | 0 | | 0 | 0 | | 0 | 0 | 0 | -1 | 1 |
| **D. Regulation concerning food marketing toward children** | **High** | -1 | 1 | 0 | 0 | -1 | 1 |  |  | 0 | 0 | 0 | | 0 | 1 | | -1 | 0 | | 0 | 0 | | 0 | 0 | 0 | 0 | 0 |
|  | **Low** | 2 | -2 | 0 | 0 | 1 | -1 |  |  | 0 | 0 | 0 | | 0 | -2 | | 2 | 0 | | 0 | 0 | | 0 | 0 | 0 | -2 | 2 |
| **E. Sugar sweetened beverage/ processed food tax** | **High** | -1 | 1 | 0 | 0 | -1 | 1 | 2 | -2 |  |  | 0 | | 0 | 0 | | 0 | 0 | | 0 | 0 | | 0 | 0 | 0 | 1 | -1 |
|  | **Low** | 0 | 0 | 0 | 0 | 0 | 0 | -2 | 2 |  |  | 0 | | 0 | 0 | | 0 | 0 | | 0 | 0 | | 0 | 0 | 0 | -2 | 2 |
| **F. Amount of free time** | **High** | -1 | 1 | 1 | -1 | -2 | 2 | 0 | 0 | 0 | 0 |  | |  | 0 | | 0 | -1 | | 1 | -2 | | 2 | 0 | 0 | 0 | 0 |
|  | **Low** | 2 | -2 | 1 | -1 | 1 | -1 | 0 | 0 | 0 | 0 |  | |  | 0 | | 0 | 0 | | 0 | 1 | | -1 | 0 | 0 | 0 | 0 |
| **G. Political will for social change** | **High** | -1 | 1 | 0 | 0 | -1 | 1 | -1 | 1 | 1 | -1 | 0 | | 0 |  | |  | -2 | | 2 | 2 | | -2 | 2 | -2 | 0 | 0 |
|  | **Low** | 2 | -2 | 0 | 0 | 2 | -2 | 2 | -2 | -2 | 2 | -1 | | 1 |  | |  | 1 | | -1 | -1 | | 1 | -1 | 1 | 1 | -1 |
| **H. Car use** | **High** | 0 | 0 | -2 | 2 | 0 | 0 | -1 | 1 | 0 | 0 | 1 | | -1 | 1 | | -1 |  | |  | -2 | | 2 | 0 | 0 | -2 | 2 |
|  | **Low** | 0 | 0 | 1 | -1 | 0 | 0 | 2 | -2 | 0 | 0 | -2 | | 2 | -2 | | 2 |  | |  | 1 | | -1 | 1 | -1 | 1 | -1 |
| **I. Street safety** | **High** | -1 | 1 | 1 | -1 | 0 | 0 | 0 | 0 | 0 | 0 | 0 | | 0 | 0 | | 0 | 0 | | 0 |  | |  | 0 | 0 | 0 | 0 |
|  | **Low** | 0 | 0 | -2 | 2 | 0 | 0 | 0 | 0 | 0 | 0 | 0 | | 0 | 0 | | 0 | 2 | | -2 |  | |  | 0 | 0 | 0 | 0 |
| **J. Public transport subsidies** | **High** | -2 | 2 | 0 | 0 | 0 | 0 | 0 | 0 | 0 | 0 | 1 | | -1 | 0 | | 0 | 0 | | 0 | 1 | | -1 |  |  | 0 | 0 |
|  | **Low** | 0 | 0 | 0 | 0 | 0 | 0 | 0 | 0 | 0 | 0 | -1 | | 1 | -1 | | 1 | 1 | | -1 | 0 | | 0 |  |  | 0 | 0 |
| **K. Price of healthy food** | **High** | 0 | 0 | 2 | -2 | -1 | 1 | 0 | 0 | 0 | 0 | 0 | | 0 | 0 | | 0 | 0 | | 0 | 0 | | 0 | 0 | 0 |  |  |
|  | **Low** | 0 | 0 | -2 | 2 | -1 | 1 | 0 | 0 | 0 | 0 | 0 | | 0 | 0 | | 0 | 0 | | 0 | 0 | | 0 | 0 | 0 |  |  |
| Scenario states**:** | |  |  |  |  |  |  |  |  |  |  |  | |  |  | |  |  | |  |  | |  |  |  |  |  |
| Impact balances: | | -6 | 6 | 1 | -1 | -6 | 6 | 3 | -3 | 1 | -1 | -3 | | 3 | 1 | | -1 | -5 | | 5 | 4 | | -4 | 3 | -3 | 2 | -2 |

Maximum impact:

The impact balances represent the impact of the example scenario on each column factor state. The most plausible factor states i.e., factor states with the highest impact scores, are highlighted by the black arrows. Finally, to determine whether the example scenario is consistent, these arrows are compared to the blue arrows, which characterize the example scenario. For the scenario to be considered consistent, the blue and the black arrow markings must correspond perfectly. In the case of the example scenario, there are three inconsistencies which means that the scenario is inconsistent.
